# Integration and Segregation of Dynamic Functional Connectivity States for Mild Cognitive Impairment Revealed by Graph Theory Indicators

**DOI:** 10.1155/2021/6890024

**Published:** 2021-07-17

**Authors:** Zhuqing Jiao, Peng Gao, Yixin Ji, Haifeng Shi

**Affiliations:** ^1^School of Computer Science and Artificial Intelligence, Changzhou University, Changzhou 213164, China; ^2^School of Microelectronics and Control Engineering, Changzhou University, Changzhou 213164, China; ^3^Department of Radiology, Changzhou Second People's Hospital Affiliated to Nanjing Medical University, Changzhou 213003, China

## Abstract

Mild cognitive impairment (MCI) is an intermediate stage between normal aging and dementia. Researchers tend to discuss its early state (early MCI, eMCI) due to its high conversion rate of dementia and poor treatment effect in the middle and late stages. Currently, the research on the disease evolution of the brain functional networks of patients with MCI has gradually become a research hotspot. In this study, we compare the differences in dynamic functional connectivity among eMCI, late MCI (lMCI), and normal control (NC) groups, and their graph theory indicators reveal the integration and segregation of functional connectivity states. Firstly, dynamic functional network windows were constructed based on the sliding time window method, and then these window samples were clustered by *k*-means to extract the functional connectivity states. The differences in the three groups were compared by analyzing the graph theory indicators, such as the participation coefficient, module degree distribution, clustering coefficient, global efficiency, and local efficiency, which distinguish the functional connectivity states. The results reveal that the NC group has the strongest integration and segregation, followed by the eMCI group, and the lMCI group has the weakest integration and segregation. We conclude that with the aggravation of MCI, the integration and segregation of dynamic functional connectivity states tend to decline. The results also reflect that the lMCI group has significantly more brain functional connections in some states, such as IPL.L-MTG.R and DCG.R-SMG.L, than the eMCI group, while the lMCI group has significantly less OLF.L-SPG.L than the NC group.

## 1. Introduction

Mild cognitive impairment (MCI) is a form of cognitive impairment that occurs between normal aging and dementia. It is marked by age-related memory loss. However, it does not meet the criteria for Alzheimer's disease (AD) [1, 2]. Recent studies reported that patients diagnosed with MCI have a high probability of converting to AD, with an annual conversion rate of 6%∼25% [3]. In the research on chronic neurodegenerative diseases, many clinical cases have proved that the treatment effect of the patients with cognitive dysfunction is not satisfactory at the middle and late stages. In recent years, researchers have turned to the diagnosis and preventive intervention of its early stage [4–6]. People with early MCI (eMCI) are unaffected in their daily lives and cannot be distinguished from normal people by the naked eye [7, 8]. There are no obvious differences between their brain functional networks, and it is difficult to extract some specific features. However, the brain functional networks of patients with late MCI (lMCI) are significantly different from that of normal people. Therefore, it is of great significance to analyze the evolution of the brain functional networks from normal people to patients with early MCI and to patients with late MCI.

Spontaneous fluctuations represent the basic mechanism of nerve signals, which can be explained to a large extent by fMRI data [8, 9]. Functional connectivity is considered as a static phenomenon in most resting-state fMRI (RS-fMRI) studies. It is calculated following the statistical dependence on brain regions during the whole scanning process [3, 10]. Some studies have suggested that functional connectivity of MCI patients has significant changes compared with normal people [11]. However, multiple fMRI and electrophysiological experiments have shown that functional connectivity fluctuates within a short time range of a few seconds [11]. Currently, more and more scholars adopt the method of detecting dynamic functional connectivity [12, 13], which reflects how the functional connectivity of the brain changes over time [14]. Some studies have found that diseases can change the dynamic characteristics of functional connectivity, which can be used as a physiological index for disease research [15], and has important theoretical and practical value for the study of the dynamic characteristics of brain functional networks. Dynamic functional connectivity can more accurately represent the dynamic features of the brain [11], whereas static functional connectivity also helps to understand brain correlations.

Nowadays, the commonly used dynamic network research methods of neuroscience include sliding time window analysis, single-frame coactivation pattern analysis, and time-frequency analysis [16, 17]. Among them, the sliding time window method repeatedly evaluates the paired connections between brain regions or voxels to obtain nonoverlapping time windows of data [6], and these windows can express the dynamic characteristics of functional connectivity. The sliding time window method plays a dominant role in dynamic functional connectivity analysis at present, due to its simplicity and ability to retrieve the significant features of functional connectivity. However, the sliding time window method has some significant limitations. First, the choice of window length has long been a topic of debate. On the one hand, if the window length is too short, it will increase the risk of introducing clutter into the observed dynamic functional connectivity. On the other hand, the excessively long window will hinder the detection of time changes in the region of interest [18, 19]. Hence, the window length is generally selected between 30 s and 60 s to maintain specificity and sensitivity [20].

Dynamic functional connectivity contains relevant information about the presence of MCI, and other neuroimaging analysis methods are needed to help understand the MCI brain network. Our first aim is to compare the differences in dynamic functional connections between eMCI, lMCI, and normal control (NC) groups in the same state by static modeling. Pearson correlation coefficients and sliding time windows are used to construct the dynamic functional network, and *k*-means clustering is used to extract functional connectivity states. Our second aim is to reveal the changes in the integration and segregation of functional connectivity states as MCI aggravates. The graph theory method is used to calculate the state indicators which distinguish the functional connectivity states, while network-based statistic (NBS) is adopted to detect functional connectivity differences.

## 2. Data and Methods

### 2.1. Data Acquisition and Preprocessing

The data used in this study were from the open data set of the Alzheimer's Disease Neuroimaging Program (ADNI) (http://adni.loni.usc.edu). The fMRI data onto 160 subjects were used for the study, including 48 eMCI patients, 45 lMCI patients, and 67 NC patients.

Before collecting fMRI data, it is necessary to understand the physical state of subjects, sign the informed consent confirmation of the subjects, ask the subjects to check whether there are metal objects on the body, and remind the subjects to keep awake and not to have any conscious thinking activities [21].

In the experiment, 3.0 T Philips Achieva was used to collect brain fMRI data: function images, 24 axial slices, layer thickness = 4 mm, repeat time TR = 2000 ms, echo time TE = 35 ms, flip angle = 90°, and field of view FOV = 230 mm × 182 mm; structure images: 3D sequence layer number = 240, layer thickness = 0.6 mm, repeat time TR = 7.4 ms, echo time TE = 3.4 ms, flip angle = 8°, and field of view FOV = 250 mm × 250 mm.

We use toolbox DPARSF (Data Processing Assistant for Resting-State fMRI) in the MATLAB 2012a environment (http://rfmri.org/DPARSF) to perform format conversion and preprocessing for fMRI data [22]. The preprocessing steps include interlayer correction, spatial registration, standardization, and filtering. The filtering range is 0.01–0.08 Hz (the standardized bounding box: [−90, −126, −72; 90, 90, 108] and voxel size: [3 3 3]). Since it takes a certain amount of time for both the machine and the subjects to enter a stable state, the image data of the previous 10 time points were removed during preprocessing [23, 24]. In the process of interlayer correction, the subjects (2 subjects) with large head movement (translation > 2.5 mm, rotation > 2.5°) were removed [25].

### 2.2. Overview of the Proposed Framework

**Figure 1:**
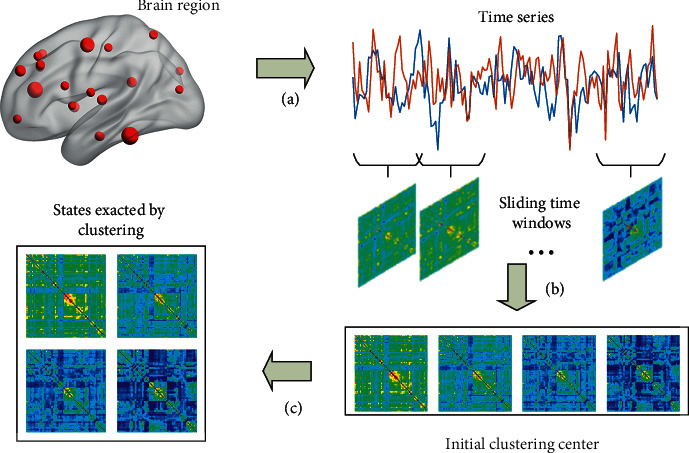
Flowchart of functional connectivity state extraction: (a) extracting the time series; (b) calculating the functional connectivity matrix within each sliding window; (c) using the *k*-means clustering for the windowed functional connectivity matrices to extract different functional connectivity states.

Figure 1 shows the flowchart of functional connectivity state extraction. The dynamic brain functional networks are constructed based on the preprocessed time series, and the functional connectivity states are extracted by using two-stage clustering. Next, the windowed dynamic functional connectivity matrices are clustered to extract the initial clustering center. Then, the initial clustering centers are taken as the parameters of the *k*-means clustering to continue the clustering of the windowed matrices. It is worth noting that the windows of the clustering center obtained at this time are only the initial clustering centers, not the final clustering results. The specific steps are as follows:

- Extract the time series of the three groups of subjects. A time series matrix of *L∗M* will be generated for each subject, where *L* is the length of the time series and *M* (*M* = 90) is the number of brain regions.
- Select *n* windows with the largest variance from *N* windows of the time series of each subject as the clustering windows, based on the sliding time window method, and place them into the same group. Cluster all windowed functional connectivity matrices in the group to extract *k* cluster center windows using *k*-means clustering.
- Cluster all windowed matrices with *k* initial clustering centers to extract functional connectivity states.

### 2.3. Clustering Analysis

The sliding time window method was used to construct the network to analyze the dynamic brain functional connectivity [4]. First, the average time series of *M* brain regions of interest (ROIs) is extracted, and then the average time series are repeatedly moved with a certain step length by using the time windows, and the correlation coefficient between brain regions is calculated each time to obtain a group of dynamic functional connectivity matrices [18, 19]. Specially, {*S*_*i*_(*t*), *t=*0,1,…,*N*, *i=*1,2,…,*M*} is used to represent the time series, where *t* is the moment and *i* is the brain region. The correlation coefficient *r* is close to −1 or 1 when Pearson correlation coefficient is employed as functional connectivity. Thus, the increment of variance will become smaller, which affects the efficiency of analysis. We carried out Fisher R-Z transformation on the correlation coefficient to make it follow the normal distribution in order to stabilize the variance [4]:

(1) $$\begin{array}{c} FZ\left(r_{ij}\left(s\right)\right)≜\frac{1}{2}\ln\left(\frac{1+r_{ij}\left(s\right)}{1-r_{ij}\left(s\right)}\right), \end{array}$$

where *r*_*ij*_(*s*) is the Pearson correlation coefficient between regions of interest *i* and *j* at time s, and *FZ*(*·*) represents Fisher R-Z transformation.

*k*-means clustering is used to identify short-term recurring connection patterns, which we describe as functional connectivity states. Functional connectivity states are predicted by models of large-scale neural connectivity. The *k*-means algorithm is an unsupervised clustering algorithm, which is widely used because of its simplicity and accuracy. For a given sample set, the samples are divided into *k* clusters according to the distance between the samples. The nodes within the cluster are connected as closely as possible, and the distances between the clusters are as large as possible. Therefore, the goal of the *k*-means algorithm is to minimize the square error *E* [24–26]:

(2) $$\begin{array}{c} \mathrm{E=}\sum_{\mathrm{i=}1}^{k}\sum_{x\in C_{i}}{\left\|x-\mu_{i}\right\|}_{2}^{2}, \end{array}$$

where *x* is all the sample vectors in the sample set, *C*_*i*_ is the sample set whose sample vectors belong to the *i*th class, and *μ*_*i*_ is the mean vector of the cluster, which is also called as the center of mass. Its expression is

(3) $$\begin{array}{c} \mu_{i}=\frac{1}{\left|C_{i}\right|}\sum_{x\in C_{i}}x. \end{array}$$

The *k*-means ++ algorithm is used to repeat *k*-means clustering for several times to avoid local minimum [27]. Since the sample is high-dimensional data, *L*1 distance function (Manhattan distance) has a more effective similarity measure than *L*2 distance function (Euclidean distance), so we adopt *L*1 distance function [28]. The Manhattan distance formula is as follows:

(4) $$\begin{array}{c} c=\left|x_{i}-x_{j}\right|+\left|y_{i}-y_{j}\right|, \end{array}$$

where *c* is the Manhattan distance, *x*_*i*_ and *y*_*i*_ are the coordinate of the node *i* in the plane, and *x*_*j*_ and *y*_*j*_ are the coordinate of the node *j*.

The elbow rule of the clustering effectiveness index is used to determine the cluster number (*k*) for group-level clustering and subject-level clustering, and the calculation methods are used to determine the ratio of intracluster distance to intercluster distance [28, 29]. The basic idea of the elbow rule is that with the increase of the cluster number *k*, the sample division will be more refined, the degree of integration of each cluster will be enhanced, and the square error *E* will gradually become smaller. When the *k* value is less than the true clustering value, the increase of *k* will greatly increase the degree of integration of each cluster, so that *E* will greatly decrease. On the contrary, if the *k* value is greater than the true clustering value, so that the sample division is too refined and the decline of *E* is small. Resampling can not only reduce the redundancy of the functional connectivity matrix but also reduce the amount of calculation [30, 31]. Like EEG microstate analysis [17, 32], we select 6–8 windows with the largest variance as samples for each subject. The *k*-means algorithm (randomly initializing the center node) was used to cluster these test samples, and the clustering was performed several times to avoid the local minimum as much as possible. The resulting center nodes are used as the initial center nodes for *k*-means clustering of all windows. Compared with randomly selecting the center nodes, selecting the center nodes through resampling has a better effect on clustering.

### 2.4. Functional Connectivity States

The temporal characteristics of dynamic functional connectivity states are studied by calculating the mean dwelling time and the number of transitions from one state to another. Mean dwelling time is defined as the number of successive windows of a state, and the number of transitions between states represents the stability of a state. Functional connectivity tends to be assigned to a single state over a long period of time, with short transitions. A two-sample *t*-test was used for detecting the group differences in mean residence time and conversion times among the normal control group, the eMCI group, and the lMCI group (*P* < 0.05) [32]. The three groups were matched for age, sex, and education.

In addition, an NBS toolbox was used to calculate the differences in functional connectivity between different groups. Brain region nodes of AAL template were used as input nodes, and *z*-scores of correlation coefficients were used as input edges. The toolbox uses a permutation test to randomly swap the group to which each subject belongs and retests it in each permutation to confirm the null hypothesis.

Brain connectivity toolbox (https://sites.google.com/site/bctnet/) is applied to the analysis of network graphic feature (global and local). The sparsity threshold needs to be fixed in order to ensure the same range of edges in graphs from different groups [33]. The sparsity value is defined as the number of connections between nodes in the network divided by the total number of possible connections in the network.

Typical indicators of brain network segregation are average clustering coefficient, modularity, etc. Clustering coefficient is defined as the number of triangles around a single node; that is, two adjacent nodes of a node are adjacent nodes to each other, which expresses the universality of clustering connectivity around each node [34]. It is usually used to describe the functional isolation of network local information processing [35], reflecting the clustering tightness of the adjacent nodes of each node [36]. Modularity is a more complex measure of network separation, which not only describes densely interconnected regional groups but also finds out the size and composition of these individual groups [37]:

(5) $$\begin{array}{c} \mathrm{CC}=\frac{1}{n}\sum_{i\in N}CC_{i}=\frac{1}{n}\sum_{i\in N}\frac{2t_{i}}{k_{i}\left(k_{i}-1\right)}, \end{array}$$

where *N* is the data set of all nodes in the network and *n* is the number of nodes. *CC*_*i*_ is the clustering coefficient of node *i*, *t*_*i*_ is the number of triangles around node *i*, and *k*_*i*_ is the degree of node *i*.

Typical indicators of brain network integration are average shortest path length and global efficiency. As shown in formula (6), the average shortest path length is defined as the average distance between any two nodes, and it is the most common method to measure network integration. Global efficiency is defined as the efficiency with which information is transmitted throughout the network. Since paths between disconnected nodes are considered infinite and the efficiency is zero, so global efficiency makes sense in disconnected networks [38]:

(6) $$\begin{array}{c} L=\frac{1}{n}\sum_{i\in N}L_{i}=\frac{1}{n}\sum_{i\in N}\frac{\sum_{j\in N,j\neq i}d_{ij}}{n-1}, \end{array}$$

where *L* is the average shortest path length, *L*_*i*_ is the average distance between node *i* and all other nodes, and *d*_*ij*_ is the distance between node *i* and node *j*.

A common indicator of centrality of brain networks is degree. Degree has a direct neurobiological explanation: a node with a higher degree interacts structurally or functionally with many other nodes. In a modular network, degree-based measures of intramodule and intermodule connections help to group nodes into different groups. The within-module degree *z*-score is the localized intramodule version of degree centrality [39], and the participation coefficient (intermodule connections) evaluates the number of intermodule connections of a single node. On one hand, the nodes with high within-module degree *z*-score but low participation coefficient are called provincial hubs, which play a role in promoting module segregation. On the other hand, as connectors hubs, the nodes with a high participation coefficient can promote global integration. Local efficiency is defined as the efficiency of transferring information from one node to other adjacent nodes [14, 17], as shown in the following formula:

(7) $$\begin{array}{c} E_{\mathrm{loc}}=\frac{1}{n}\sum_{i\in N}E_{\mathrm{loc},i}=\frac{1}{n}\sum_{i\in N}\frac{{\sum_{j,h\in N,j\neq i}a_{ij}a_{ih}\left[d_{jh}\left(N_{i}\right)\right]}^{-1}}{k_{i}\left(k_{i}-1\right)}, \end{array}$$

where *E*_loc,*i*_ is the local efficiency of node *i*, *a*_*ij*_ is the connectivity state between node *i* and node *j*, and *a*_*ij*_ is 1 when there is a connection between node *i* and *j*; when there is no connection between node *i* and *j*, *a*_*ij*_ is 0. *d*_*jh*_(*N*_*i*_) is the shortest path length between node *j* and node *h*.

## 3. Experiments and Results

### 3.1. Construct Functional Connectivity State

**Figure 2:**
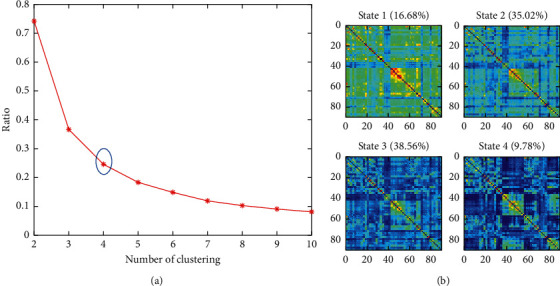
Brain function connectivity states. (a) The elbow rule is used to determine the number of functional connectivity state. (b) Visualization results of functional connectivity states formed by clustering brain functional network windows in the NC group, the eMCI group, and the lMCI group.

Figure 2 shows the state number and state visualization results of constructing functional connectivity states. As shown in Figure 2(a), the ratio of intraclass distance to interclass distance keeps changing with the clustering number *k* from 2 to 10. The vertical axis of the image decreases slowly, which meets the requirement of the elbow rule to determine the cluster numbers when *k* = 4. Accordingly, we select *k* = 4 as the cluster number.  Figure 2(b) shows the result of clustering analysis of eMCI, lMCI, and NC groups. As can be seen, four matrices represent four functional connectivity states. Each state represents the center of mass of the cluster and reflects the pattern that exists stably in the data set. We use the resampling method to improve the efficiency of the experiment. Among them, both State 2 and State 3 accounted for higher proportions, 35.02% and 38.56%, respectively.  Table 1 shows the analysis of the basic indicators of the four states, and the states are sorted from the highest mean value to lowest. All four states have small standard deviations. Negative connectivity is present in States 3 and 4.

**Figure 3:**
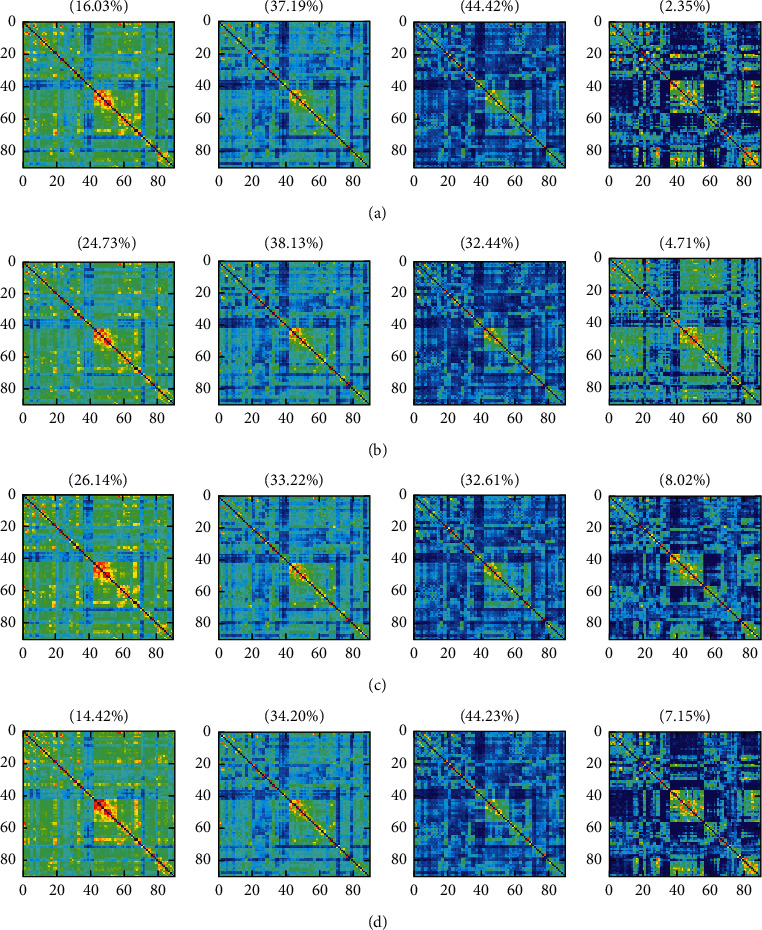
Clustering results of bootstrap resample experiments. (a–d) The clustering results of four bootstrap resample experiments, respectively.

There were 160 subjects performed multiple-bootstrap resample in order to verify the validity of these states. Bootstrap resample is used to verify the accuracy and uncertainty of the prediction model. It randomly selects a few samples of observed values from the original data set to evaluate the model [19]. The clustering result in Figure 2(b) is repeated in the bootstrap resample for several times [4], and the proportion of the number of states in the multiple resampling shows no significant changes. As shown in Figure 3, States 1 to 4 of the bootstrap resample are ranked from the highest to the lowest mean value. The clustering results of each bootstrap resample are displayed for each row. Among them, the average value of the first state reaches 0.6, the average value of the last state is around 0.2, and the sum of percentage of the appearance times of State 2 and State 3 reaches more than 70%. The results like the states in Figure 2(b) have appeared many times in the sampling experiment of bootstrap resample, which can be proved that the states we extracted are highly accurate.

**Figure 4:**
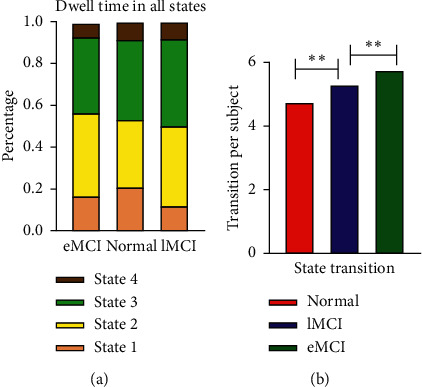
Dynamic function connectivity state differences. (a) Ratio of dwelling time of each state in the eMCI group, lMCI group, and NC group. (b) The differences of state transition among the three groups. ^*∗∗*^ Significant differences between two groups.

Figure 4 shows the differences in the functional connectivity states of three groups of subjects.  Figure 4(a) shows the proportion of three groups of subjects' dwelling time in each state. The proportion of three groups of subjects' dwelling time in State 2 and State 3 is both more than 0.3, followed by State 1, and State 4 takes up the least proportion. The proportion of State 1 in the NC group is higher than that in the other two groups, while the proportion of State 2 is lower. The proportion of State 3 of the lMCI group is significantly higher than that in the other two groups.  Figure 4(b) shows the differences between the state transitions of three groups of subjects. The NC group, the lMCI group, and the eMCI group were ranked from the lowest to the highest mean times of transition of each subject. The double-sample *t*-test was conducted for the mean times of transition of the three groups. The times of transition are significantly different from the NC group and the lMCI group (*t* = 8.138, *P* < 0.01), and the times of transition are also significantly different from the eMCI group and the lMCI group (*t* = 5.479, *P* < 0.01). There is no significant difference for transition times between the control group and the eMCI group (*t* = 1.49, *P*=0.14).

**Figure 5:**
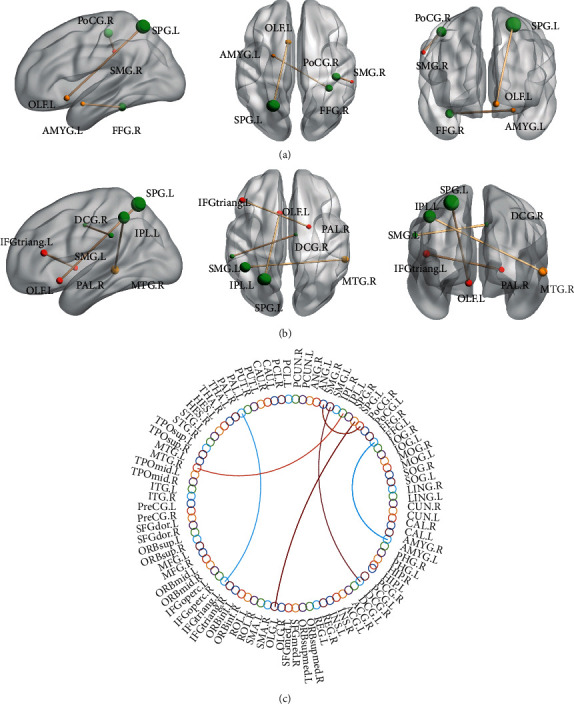
Brain functional connections in eMCI, lMCI, and NC groups have significant differences. (a) The tripartite view with significant differences in functional connections between groups in State 2. OLF.L-SPG.L is the functional connection with significant difference between the NC group and the lMCI group, while the remaining connections are the functional connections with significant differences between the NC group and the eMCI group. (b) The tripartite view with significant differences in functional connections among groups in State 3. IPL.L-MTG.R and DCG.R-SMG.L are the functional connections with significant differences between the eMCI group and the lMCI group, and OLF.L-SPG.L is the functional connection with significant differences between the NC group and the lMCI group. IFGtriang.L-PAL.R is the functional connection with significant differences between the NC group and the eMCI group. (c) A circular chart of the functional connections that differ significantly between groups.

In addition, the NBS method is used to calculate the differences in functional connectivity among different groups in the same state. Considering the high proportion of dwelling time for State 2 and State 3, the functional connections in the two states are compared. As shown in Figure 5, in State 2, the functional connection of the lMCI group: OLF.L-SPG.L (*t* = 3.87, *P* < 0.05, *P* value corrected by FDR) is significantly lower than that of the NC group. The functional connections POCG.R-SMG.R (*t* = 3.71, *P* < 0.05) and AMYG.L-FFG.R (*t* = 3.9, *P* < 0.05) of the eMCI group are significantly lower than those of the NC group. In State 3, there are significant differences in functional connections between the eMCI group and the lMCI group: IPL.L-MTG.R (*t* = 3.51, *P* < 0.05) and DCG.R-SMG.L (*t* = 4.56, *P* < 0.05), and the functional connections in eMCI group are significantly less than the lMCI group. IFGtriang.L-PAL.R (*t* = 4.42, *P* < 0.05) of the NC group is significantly more than those of the eMCI group. The experimental results indicate that in States 2 and 3, both the eMCI group and the lMCI group have significantly less functional connections than the NC group, while the lMCI group also has significantly more functional connections than the eMCI group.

### 3.2. Analyze State Indicators and Module Partition Results

**Figure 6:**
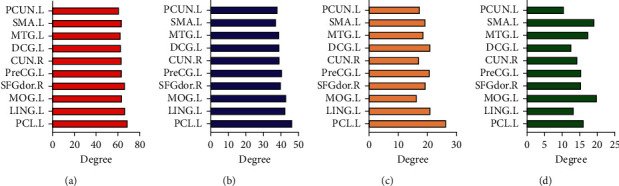
Degrees of important node in different states. (a) State 1, (b) State 2, (c) State 3, and (d) State 4.

The brain functional connections lower than 0.3 are regarded as disconnected connections when calculating these graph theory indicators, and the default value is 0.  Figure 6 shows the top 20 brain regions or regions of interest about node degree of each state. Ten brain regions, PCUN.L, SMA.L, MTG.L, DCG.L, CUN.R, PreCG.L, SFGDOR.R, MOG.L, LING.L, and PCL.L, were found to rank high in four states. The degree of a single node is equal to the sum of the weights of connections connected to the node, and the degree value represents the centrality of the node in the network [1]. As can be seen from the figure, the degree value shows a downward trend from State 1 to State 4. The degree value of the top nodes in State 1 reaches 60, while the degree value of these nodes in State 4 is about 15.

**Figure 7:**
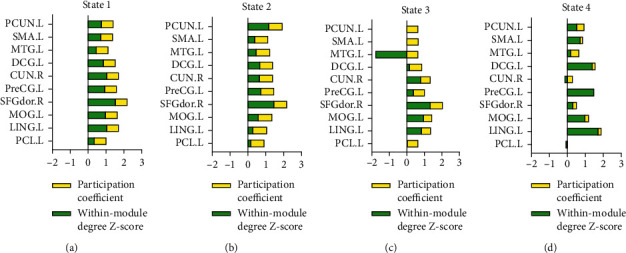
Participation coefficient and within-module degree *z*-score of important nodes in different states. In (a), each node has the highest within-module degree *z*-score and high participation coefficient. (b) is similar to (a). The participation coefficients of (c) and (d) are significantly reduced, and the within-module degree *z*-score of (c) is the lowest.

We calculated the participation coefficients and within-module degree *z*-score in each state in order to analyze the role of these nodes in the dynamic brain functional network. As shown in Figure 7, the participation coefficient in State 1 is the highest and the within-module degree *z*-score is also high. This phenomenon indicates that these nodes play the role of connector hubs in the brain functional network, which in turn leads to a strong integration of the functional connectivity states. In State 2, nodes' within-module degree *z*-score decreases, indicating a decreased integration. In State 3, the within-module *z*-score and the participation coefficient are significantly decreased, indicating a decreased integration and an enhanced segregation. In contrast, the participation coefficient of State 4 further decreases, while the within-module degree z-score significantly increases. It indicates that these nodes have begun to play the role of provincial hubs in the network.

Table 2 shows the proportions of the eMCI group, NC group, and lMCI group in different states. It is not difficult to find that the proportions of the three groups of subjects in States 2 and 3 are higher, while the proportions in States 1 and 4 are lower. The differences in the three groups are mainly the number of State 1 and State 2. The proportion of State 1 of the NC group is the highest (0.2053), followed by the eMCI group and the lowest (0.1169) of the lMCI group. Moreover, the proportion of the eMCI group in State 2 is the highest (0.3833), followed by the lMCI group, and the lowest of the NC group (0.3175). It is hereby inferred that the proportion of States 1 and 2 changed significantly during the disease evolution.

**Figure 8:**
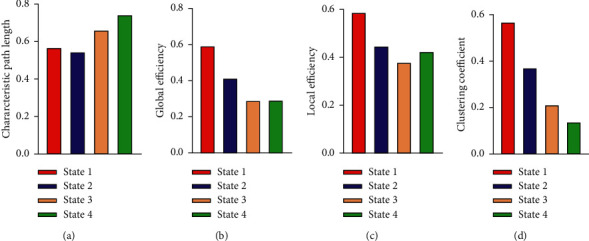
Global indicators of functional connectivity states. (a) The characteristic path length of the four states, (b) the global efficiency of the four states, (c) the local efficiency of the states, and (d) the clustering coefficient of the states.

Figure 8 shows the analysis of global indicators of functional connectivity states, including global efficiency, local efficiency, clustering coefficient, and shortest path length [40]. As can be seen from Figure 8, the characteristic path length decreases with the decrease of state mean value, while the local efficiency, global efficiency, and clustering coefficient increase from State 1 to State 4. The integration and segregation from State 1 to State 4 became weaker and weaker according to the measurement of integration and segregation proposed by Rubinov [1]. It reveals that the NC group has the strongest integration and segregation of functional connectivity states, followed by the eMCI group and the lMCI group, considering that the number of State 1 of the NC group is the highest as well as that of State 3 of the lMCI group among the three groups.

### 3.3. Select Window Length Parameters

**Figure 9:**
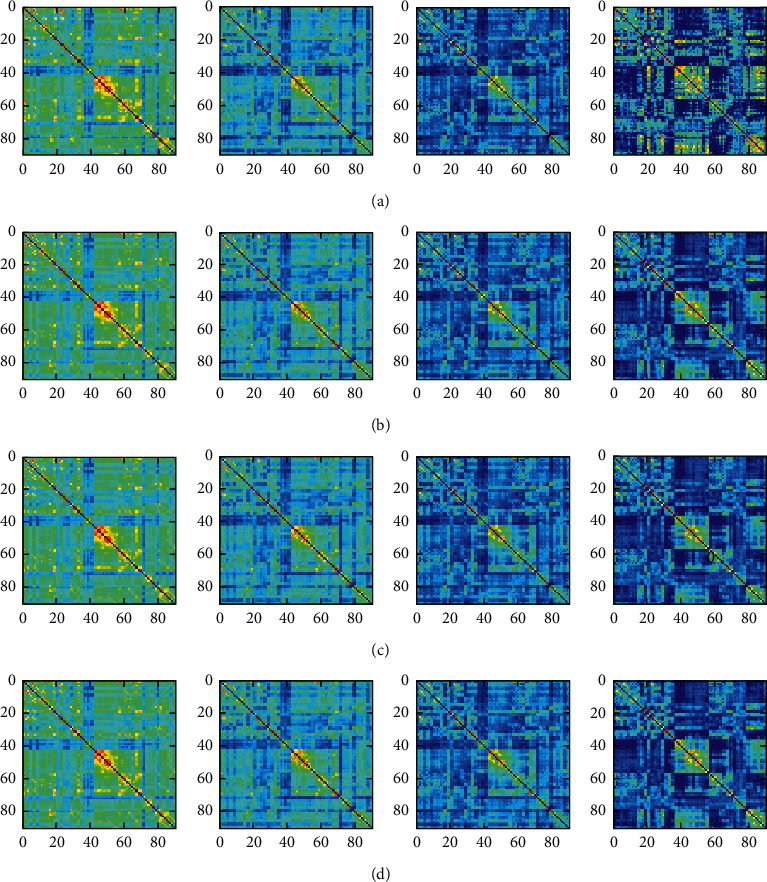
Correlation matrices of functional connectivity states extracted by different window lengths. (a–d) are the correlation matrices of functional connectivity states formed when the window length is 30 s, 40 s, 50 s, and 60 s, respectively.

Figure 9 shows that the window length selected in 30–60 s. 30 s, 40 s, 50 s, and 60 s is used as variables to calculate the four states about functional connections, respectively [13]. As can be seen from the figure, differences in the functional network constructed by different window lengths are not obvious. Then, we get similar results by calculating the mean value, standard deviation, and maximum and minimum value of each state of each window length (see Table 3). We believe that the mean value and variance of the brain functional connection network constructed by this window length (30 s) are large enough for the subsequent analysis and research when 30 s is selected as the window length. A large number of windows ensure sufficient data for the experiment.

## 4. Discussion

In summary, the dynamic brain functional networks are constructed based on the sliding time window method, and the correlation matrices of few substates are extracted by clustering these networks twice. Compared with the random selection of the initial clustering centers, this method performs one more clustering and adds some computational complexity, but it significantly improves the accuracy of clustering. In addition, on this basis, the differences in brain functional networks of eMCI, lMCI and NC groups are compared through graph theory analysis of these states. The results demonstrate that there are significant differences in brain functional connections between the lMCI group and the NC group, while there are no significant differences in the brain functional connectivity between the eMCI group and the NC group.

Firstly, there are 160 subjects resampled by the bootstrap resample method. After clustering these resampled samples, we found that their clustering results were very similar to the previous clustering results, which strongly proved the accuracy of clustering results. The whole sample presents four different connectivity states, which are ranked in order of mean value. What these three groups have in common is there is a high proportion of State 2 and State 3 and a low proportion of State 4.

Secondly, the average dwelling time and the number of state transitions of three groups are calculated. It is found that the NC group stays longer in State 1, while the lMCI group stays longer in State 3. The number of state transitions in the NC group is the least, while there are a lot of transitions in lMCI and eMCI groups. This indicates that the functional connectivity states of the NC group are stable, while the functional connectivity states of MCI patients fluctuate greatly. Previous studies [30] have proved that changes in dynamic functional connections may be related to the performance of cognitive ability, and the results of this paper confirm this inference. Graph theory analysis is carried out with the indicators such as within-module degree *z*-score, participation coefficient, clustering coefficient, and characteristic path length, to explore the differences in these states. The results show that the node degrees of the main nodes whose degrees rank high in all nodes from State 1 to State 4 decrease continuously. These high-degree nodes are important to the whole network because they play a key role in transmitting information. In addition, the main nodes in State 1 play the role of the core of the network due to the high within-module degree *z*-score and participation coefficient, while the main nodes in State 3 have weak integration and segregation of functional connectivity.

Meanwhile, we conduct a statistical test of functional connections among groups in order to study the differences in brain functional connections. The results indicate that OLF.L-SPG.L is absent from the lMCI group, while POCG.R-SMG.R and AMYG.L-FFG.R are absent from the eMCI group in State 2. OLF.L-SPG.L is absent from the lMCI group and IFGtraiang.L-PAL.R is absent from the eMCI group in State 3. This suggests that patients with MCI have some reduced functional connections, which in turn affects the connectivity of the entire brain's functional network.

In practice, some limitations of the study must be considered. We did not find biomarkers that differentiated patients with eMCI from normal people, although finding reasonable biomarkers is more helpful for the following classification. In the field of dynamic brain connectivity, the determination of reasonable functional connectivity states is a key and controversial issue. The development and improvement to more effective methods of recognition of connectivity states will be more conducive to the understanding of the pathophysiological mechanisms of mental diseases. In addition, our sample size is relatively small, which cannot fully represent the abnormalities of functional networks of patients with large samples [17, 40, 41]. In future work, we will focus on finding biomarkers that can be used to classify patients with eMCI from normal people in dynamic functional network. In addition, we will also try to adopt structural data to research and analysis in further exploring the brain network of MCI patients [42–44].

## 5. Conclusion

We examined and analyzed various graph theory indicators of functional connectivity states of the eMCI group, the lMCI group, and the NC group. It is found that the lMCI group has smaller participation coefficients, smaller within-module degree z-scores, longer characteristic path lengths, and lower local efficiency of brain functional networks than the eMCI group and the NC group. Therefore, it is concluded that the NC group has the strongest integration and segregation, followed by the eMCI group, and the lMCI group has the weakest integration and segregation [1, 45, 46]. In addition, brain functional connections in some states such as IPL.L-MTG.R and DCG.R-SMG.L of the lMCI group are significantly more than those of the eMCI group, and OLF.L-SPG.L of the lMCI group is significantly less than those of the NC group. Exploring these abnormal connections can help us better understand the differences in eMCI, lMCI, and NC groups [47–49]. In future work, we plan to investigate the changes of brain structural connectivity in patients with eMCI and lMCI [50, 51], which will improve our understanding of dynamic brain connectivity. Furthermore, relevant methods will provide enlightenment for an explainable diagnosis of cognitive impairment caused by COVID-19 [52–54].

## Figures and Tables

**Table 1:** The indicators of the four functional connectivity states clustered by *k*-means.

	State 1	State 2	State 3	State 4
Mean	0.5824	0.3925	0.2470	0.2002
Standard deviation	0.0474	0.0366	0.0323	0.0568
Maximum	2.0263	1.7329	1.5108	1.6001
Minimum	0	0	−0.1094	−0.4471

**Table 2:** The proportion of different states in the three groups.

	State 1	State 2	State 3	State 4
eMCI	0.1612	0.3833	0.3737	0.0818
Normal	0.2053	0.3175	0.3816	0.0956
lMCI	0.1169	0.3634	0.4033	0.1165

**Table 3:** Characteristics of functional connectivity states constructed by different window lengths.

	Window length (s)	State 1	State 2	State 3	State 4
Mean	30 s	0.5824	0.3925	0.2470	0.2002
	40 s	0.5605	0.3858	0.2484	0.1984
	50 s	0.5523	0.3875	0.2536	0.1957
	60 s	0.5554	0.3954	0.2667	0.1935

Standard deviation	30 s	0.0474	0.0366	0.0323	0.0568
	40 s	0.0462	0.0366	0.0320	0.0584
	50 s	0.0452	0.0369	0.0322	0.0577
	60 s	0.0449	0.0381	0.0324	0.0488

Maximum	30 s	2.0263	1.7329	1.5108	1.6001
	40 s	1.9935	1.7233	1.5120	1.6123
	50 s	1.9803	1.7315	1.5141	1.6194
	60 s	1.9938	1.7429	1.5289	1.5827

Minimum	30 s	0	0	−0.1094	−0.4471
	40 s	0	0	−0.1148	−0.4562
	50 s	0	0	−0.1102	−0.4488
	60 s	0	−0.0019	−0.1013	−0.3586

## Data Availability

The data used in our experiments are available at http://adni.loni.usc.edu.
